# Improving the maize crop row navigation line recognition method of YOLOX

**DOI:** 10.3389/fpls.2024.1338228

**Published:** 2024-03-28

**Authors:** Hailiang Gong, Weidong Zhuang, Xi Wang

**Affiliations:** College of Engineering, Heilongjiang Bayi Agricultural University, Daqing, China

**Keywords:** navigation lines, maize inter-row weeding, attention mechanisms, YOLOX-Tiny, loss function

## Abstract

The accurate identification of maize crop row navigation lines is crucial for the navigation of intelligent weeding machinery, yet it faces significant challenges due to lighting variations and complex environments. This study proposes an optimized version of the YOLOX-Tiny single-stage detection network model for accurately identifying maize crop row navigation lines. It incorporates adaptive illumination adjustment and multi-scale prediction to enhance dense target detection. Visual attention mechanisms, including Efficient Channel Attention and Cooperative Attention modules, are introduced to better extract maize features. A Fast Spatial Pyramid Pooling module is incorporated to improve target localization accuracy. The Coordinate Intersection over Union loss function is used to further enhance detection accuracy. Experimental results demonstrate that the improved YOLOX-Tiny model achieves an average precision of 92.2 %, with a detection time of 15.6 milliseconds. This represents a 16.4 % improvement over the original model while maintaining high accuracy. The proposed model has a reduced size of 18.6 MB, representing a 7.1 % reduction. It also incorporates the least squares method for accurately fitting crop rows. The model showcases efficiency in processing large amounts of data, achieving a comprehensive fitting time of 42 milliseconds and an average angular error of 0.59°. The improved YOLOX-Tiny model offers substantial support for the navigation of intelligent weeding machinery in practical applications, contributing to increased agricultural productivity and reduced usage of chemical herbicides.

## 1 Introduction

In contemporary agriculture, the cultivation and management of maize are intricately linked with advancements in agricultural weed control machinery (Flores et al., 2020). Intelligent weed control machinery has become indispensable for the optimal growth of maize crops (Dhanaraju et al., 2022; Balaska et al., 2023). Accurate identification of navigation lines in maize crop rows enables precise navigation of intelligent weed control machinery, allowing for automatic control to avoid seedling damage (Machleb et al., 2020; Vrochidou et al., 2022). This capability not only mitigates the labor-intensive process of manual weeding, thereby enhancing work efficiency, but also significantly improves field management practices. However, the task of accurately identifying these navigation lines is fraught with challenges such as low illumination, object occlusion, and camera motion, which can lead to false and missed detections, thus undermining the accuracy of navigation line identification. Therefore, it is of great significance to develop a robust and accurate crop row detection algorithm for maize seedlings in complex lighting conditions.

The advent of Convolutional Neural Networks (CNNs) has marked a significant milestone in the field, revolutionizing the detection of weeds and crop rows under a wide array of conditions with its robust performance (Hu et al., 2024). While CNNs have demonstrated formidable capabilities in feature extraction and classification, the fundamental principles of simpler methods such as edge detection and shape analysis continue to offer valuable insights for enhancing algorithmic efficiency and adaptability. Prior to the emergence of CNNs, these simpler, non-CNN-based methodologies laid the groundwork, providing foundational insights and techniques that still guide current research and applications. These studies underscore the enduring relevance of simpler, non-CNN-based approaches in the ongoing evolution of agricultural image processing applications (Coleman et al., 2022). Traditional methods have employed a diverse array of image processing techniques. Ranging from methods based on the Hough Transform (HT), which utilizes point-line duality for geometric feature detection (Cha et al., 2006), to least squares fitting, vanishing point analysis, stereovision, and methods relying on the analysis of horizontal strips, each approach offers unique advantages in specific contexts (Yenikaya et al., 2013; Jiang et al., 2015). However, they also present limitations in computational efficiency, sensitivity to noise, and adaptability to complex field conditions such as high weed pressure or significant crop loss. Among these simpler methods, edge detection serves as a fundamental technique for identifying the boundaries and shapes of objects within images (Parra et al., 2020). has showcased the application of edge detection for weed recognition in lawns, demonstrating its potential in distinguishing between grass and weeds through the analysis of visual features. Similarly (Kazmi et al., 2015), has exploited affine invariant regions and the unique shapes of leaf edges for weed detection, emphasizing the effectiveness of geometric and morphological characteristics in differentiating between crops and weeds.

To address these challenges, Researchers have turned to Convolutional Neural Networks (CNNs) for their prowess in feature extraction (Hu et al., 2024). CNNs are adept at learning rich feature representations from raw images (Wu et al., 2020). The end-to-end training of CNNs enables object detection methods to better adapt to different object categories and complex scenes. Presently, deep learning-based object detection methods can be divided into two categories: two-stage object detection methods and one-stage object detection methods. Two-stage methods, renowned for their robustness against external environmental factors, employ a region proposal network (RPN) to generate potential object boxes, which are then refined through classification scoring and bounding box regression. Despite their impressive performance across multiple object detection tasks, these methods are hampered by slow detection speeds, limiting their applicability in real-time scenarios. In response, the agricultural sector has witnessed a surge in the integration of one-stage object detection methods, such as the You Only Look Once(YOLO) network models, into crop detection processes (Rakhmatulin et al., 2021). This integration facilitates high-accuracy, real-time row detection, laying the groundwork for precise extraction of navigation lines from crop images. Efforts to refine one-stage detection methods have led to significant advancements in detection accuracy, enabling the development of innovative solutions such as the “LettuceTrack” by (Hu et al., 2021), which employs the YOLOv5 network model for vegetable detection and tracking, and the real-time vehicle recognition and tracking method proposed by (Wang L. et al., 2023), leveraging an improved YOLOv4 model (Yang et al., 2022). utilized the YOLOv3 network model to fit seedling crop rows based on detection results. Firstly, they processed the seedling images within the detection box through grayscale filtering to segment the seedling crop and soil background. Then, they located the seedling feature points in the detection box using SUSAN corner features. Finally, they employed the least squares method to fit the seedling rows. In a recent study conducted by (Zhu et al., 2022), a weeding robot was designed and implemented for efficient weed control in maize fields. The weeding robot achieved an average detection rate of 92.45% for maize seedlings (Ruan et al., 2023). proposed a novel crop row detection method for unmanned agricultural machinery based on YOLO-R. This method incorporates the DBSCAN clustering algorithm to accurately estimate the number of crop rows present in an image, as well as the number of crops within each row (Hu and Huang, 2021). sought to address the challenges posed by weed distribution and lighting intensity on crop row detection. To achieve this, they proposed an enhanced YOLOv4 network model combined with a clustering algorithm. The algorithm exhibited reliable detection performance in scenarios with isolated weed distribution and optimal lighting conditions. However, its detection effectiveness was found to be compromised in situations with high weed pressure (Diao et al., 2023). proposed a navigation line extraction algorithm for a maize spraying robot using an improved YOLOv8s network model and the Atrous Spatial Pyramid Pooling (ASPP) technique. They located the navigation lines by extracting feature points from the maize canopy in the maize field. Their methods demonstrated higher accuracy and stability compared to other algorithms such as SUSAN corner detection and FAST corner detection.

Detecting crop rows accurately in complex field conditions, characterized by low light intensity and high weed density, presents significant challenges. To overcome these challenges, researchers have been diligently investigating novel methodologies (Wang S. et al., 2023). introduced an improved YOLOv5 network model and a centerline extraction algorithm for detecting straight and curved crop rows, specifically designed for rice seedlings. Nevertheless, this method did not consider the complexity introduced by different growth stages or environmental conditions (Yang et al., 2023). proposed a method that combines the YOLOv5 network model, hyper-green method, and Otsu method. Researchers have endeavored to tackle the intricacies involved in detecting crop rows under complex field conditions, characterized by factors such as high weed presence, dense distribution, leaf occlusion, and broken rows. To address these challenges, researchers have employed diverse methodologies. These approaches involve segmenting the crop rows and background within the region of interest, locating feature points using the FAST corner detection algorithm, and fitting the lines of maize crop rows using the least squares method (Liang et al., 2022). introduced an algorithm that combines edge detection with the Otsu method to accurately determine the contour of seedling columns in wide-row planting cotton with two narrow rows. To address challenges such as missing seedlings and furrow interference, they employed the least squares method to fit the navigation line in the gap between the two narrow rows (Wang and He, 2021). presented a robust approach for accurately detecting apple fruitlets in challenging growth environments. These environments are characterized by uncertain lighting conditions and the occlusion of apple fruits within clusters. To address these challenges, they utilized the channel pruned YOLOv5s network model. This algorithm effectively simplified the YOLOv5s model while maintaining high detection accuracy (Meng et al., 2023). have developed a spatio-temporal convolutional neural network model that leverages the Transformer architecture for the detection of pineapple fruits. Utilizing a dataset of 2,000 annotated images, the model achieves an optimal detection accuracy of 92.54% for the single-category target of pineapples. The challenge escalates as images captured from greater distances include smaller targets and a higher number of instances, complicating the detection accuracy. Despite these complexities, the model maintains high detection rates while boasting an average inference time of merely 0.163 seconds. In the realm of litchi harvesting (Wang et al., 2023), addressed the issue of branch occlusion leading to picking failures. By integrating the YOLOv8 model with both monocular and binocular image processing techniques, they investigated a method for the identification and localization of litchi picking points, incorporating a visual system for the active removal of obstructions. The precision of segmenting litchi fruits and branches reached 88.1%, with an average localization error for picking points at 2.8511mm, demonstrating a significant advancement in the automation of fruit harvesting (Fu et al., 2021). improved the YOLOv3-tiny model to enhance differentiation between kiwifruit and complex backgrounds, as well as address fruit occlusion issues. The model effectively utilized low-light information in nighttime environments, resolving the problem of insufficient lighting. Even when using small hardware devices, it achieved real-time kiwifruit detection in orchards (Zhang et al., 2023). enhanced the YOLOv4 model by integrating the lightweight neural network Mobilenetv3 and depthwise separable convolution. This modification aimed to address the limited feature extraction capability found in lightweight models. The proposed approach exhibited notable advancements in terms of detection accuracy and speed for tea canopy shoots. Extensive validation experiments conducted in tea plantation environments showcased its robust performance across a range of lighting conditions.

This study aims to further the development of intelligent recognition capabilities for crop row navigation lines in maize weeding machinery, particularly for intertillage operations. By focusing on the challenges posed by variable and complex field conditions at different growth stages, and the necessity for model adaptability during nighttime operations, we propose an optimization of the YOLOX-Tiny single-stage detection network model. This optimized, lightweight detection methodology is designed to accurately identify maize crops under various growth conditions, facilitating the extraction of corresponding navigation lines. Additionally, the reduction in model size is essential for deployment on embedded systems, ensuring practical applicability across a range of agricultural settings.

## 2 Materials and methods

This chapter provides a detailed exposition of the modifications implemented on the YOLOX model for its application in maize crop recognition tasks. Initially, Section 2.1 introduces the comprehensive experimental workflow, encompassing data collection and preprocessing, model training and optimization, as well as model deployment and application. Subsequently, Section 2.2 delves into the principal modifications made to the structure of the YOLOX-Tiny model, such as multi-scale prediction, the integration of attention mechanisms, and the incorporation of the SPPF module. Section 2.3 discusses our adoption of a Gamma image enhancement method based on Retinex theory, aimed at improving image recognition performance under varying lighting conditions. In Section 2.4, we present the evaluation results of the model on the test dataset and compare its performance with other prevalent approaches. Finally, Section 2.5 outlines our use of the least squares method for extracting navigation routes.

### 2.1 Experimental procedure

The experimental workflow is comprised of three main steps:

The first part involves the collection and processing of maize images. In this phase, meticulous preprocessing tasks are performed on the acquired maize imagery, including denoising and contrast adjustment to ensure image quality. Subsequently, image augmentation techniques are employed to expand the dataset, laying a solid foundation for model training.

The second part pertains to the training and optimization of the model. A series of innovative modifications were applied to the existing YOLOX network model, including but not limited to the introduction of a new illumination adjustment module to adapt to varying lighting conditions, the integration of an enhanced attention mechanism to improve the model’s recognition capabilities of maize plant features, and the substitution of the original loss function to optimize the training process. Given the limitations in the number of samples, data augmentation strategies were utilized. These optimization measures resulted in significant improvements in both accuracy and detection speed of the model. Furthermore, the model was comprehensively benchmarked against other leading object detection models, and its comprehensive detection capabilities were further validated by substituting different feature extraction networks.

**Figure 1:**
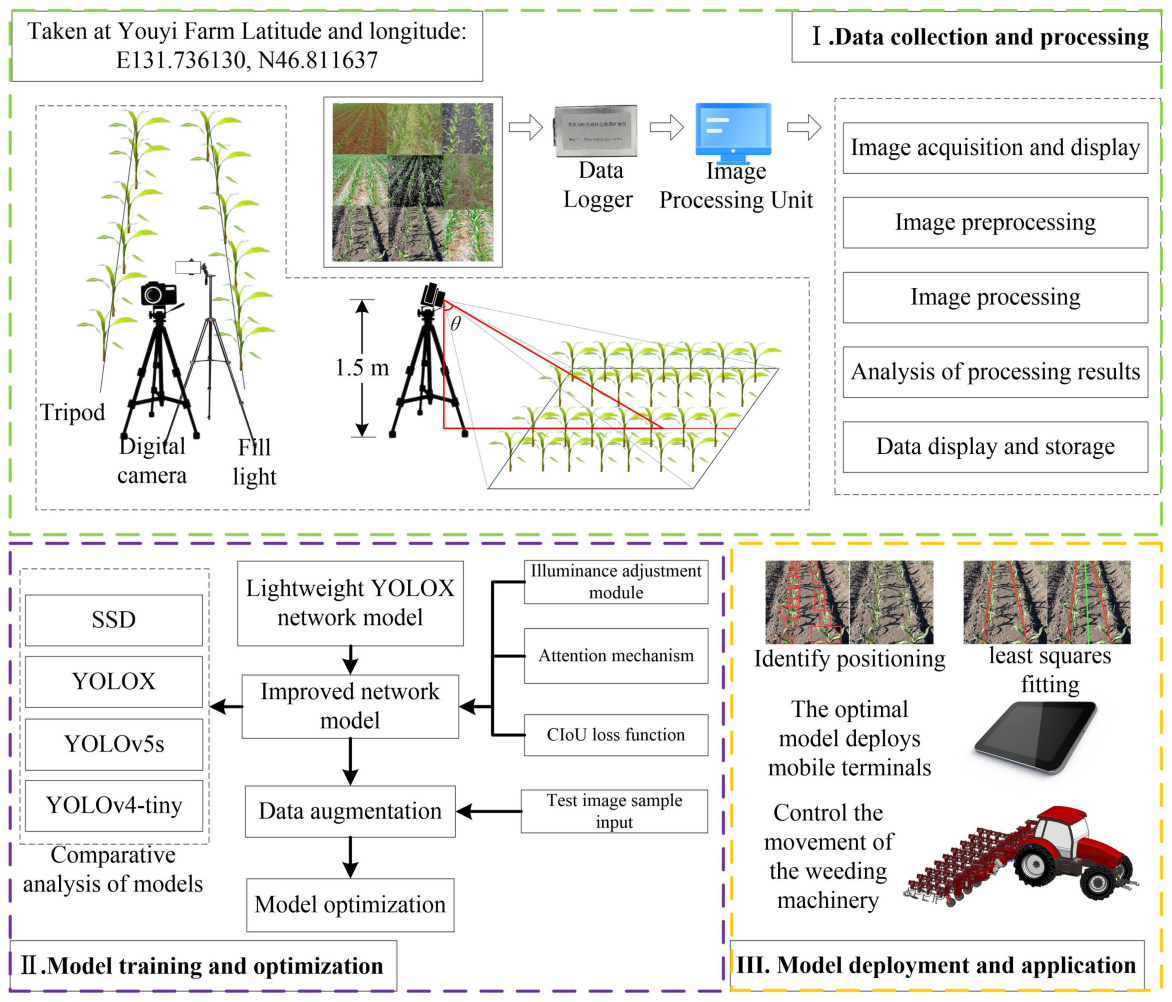
Research workflow diagram.

The third part focuses on the deployment and application of the model. In this stage, the optimized model was deployed on a mobile control terminal to achieve precise identification of maize plant positions. By fitting crop rows using the least squares method, the midline between two fitted lines was determined and used as a navigation guide to direct the weeding machinery’s path. This intelligent navigation system not only minimizes damage to maize plants but also effectively removes weeds between crop rows. After a series of validation tests, the detection algorithm was confirmed to possess the capability for efficient recognition and control operations in intelligent weeding machinery. The research workflow diagram is shown in **Figure 1**.

### 2.2 Data materials

#### 2.2.1 Data collection

**Figure 2:**
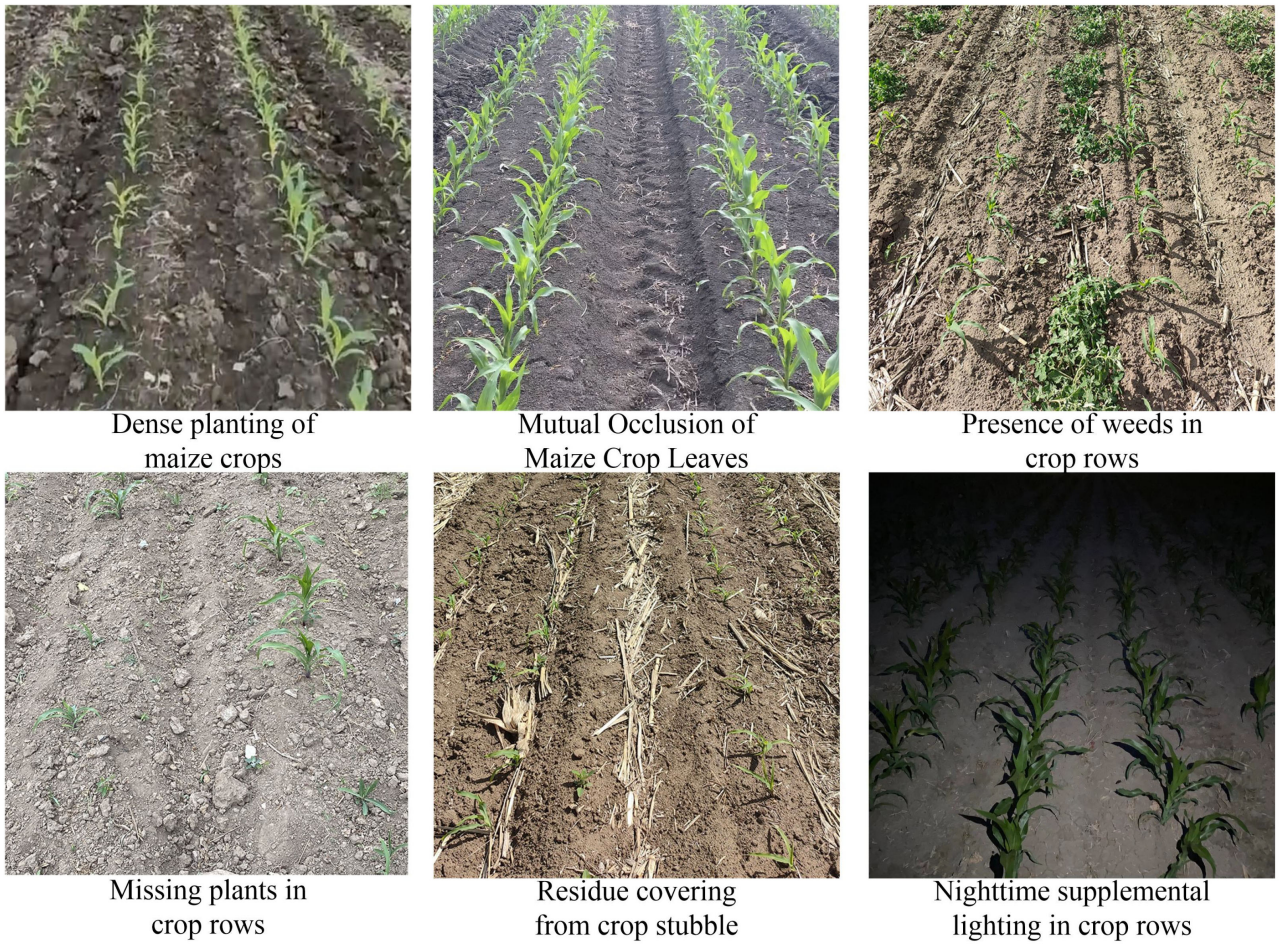
Dataset image of maize crop rows.

The prime weeding window for maize, identified as the 3-5 leaf stage, dictated the scheduling of this experiment for June 10-15, 2023. The study was conducted on plots 2-10 of the second division of Friendship Farm. Utilizing a Nikon D3100 camera, multi-angle photography of the maize fields was executed. The camera was positioned at a height of 1.5 meters, with shooting angles ranging between 30° and 60°. Photographic sessions were arranged at four distinct times: 8:00 AM, 12:00 PM, 6:00 PM, and 8:00 PM, under varying illumination conditions, culminating in a total of 800 images. The photographic process meticulously accounted for several factors including leaf occlusion, straw residue cover, plant gaps, weed interference, the dense planting of maize crops, and the application of nighttime supplemental lighting along the crop rows, as depicted in **Figure 2**.

To enhance the accuracy of crop identification under conditions of nighttime supplemental lighting, specific attention was given to capturing images with additional illumination during the night. The supplemental lighting was provided at an intensity of 1500 to 2500 lumens, installed at a height of 1.5 meters to align with the camera’s elevation. Camera settings were meticulously adjusted to an aperture of f/2.8, a shutter speed of 1/250s, and an ISO value of 200, to optimize image quality for the intended analysis.

**Figure 3:**
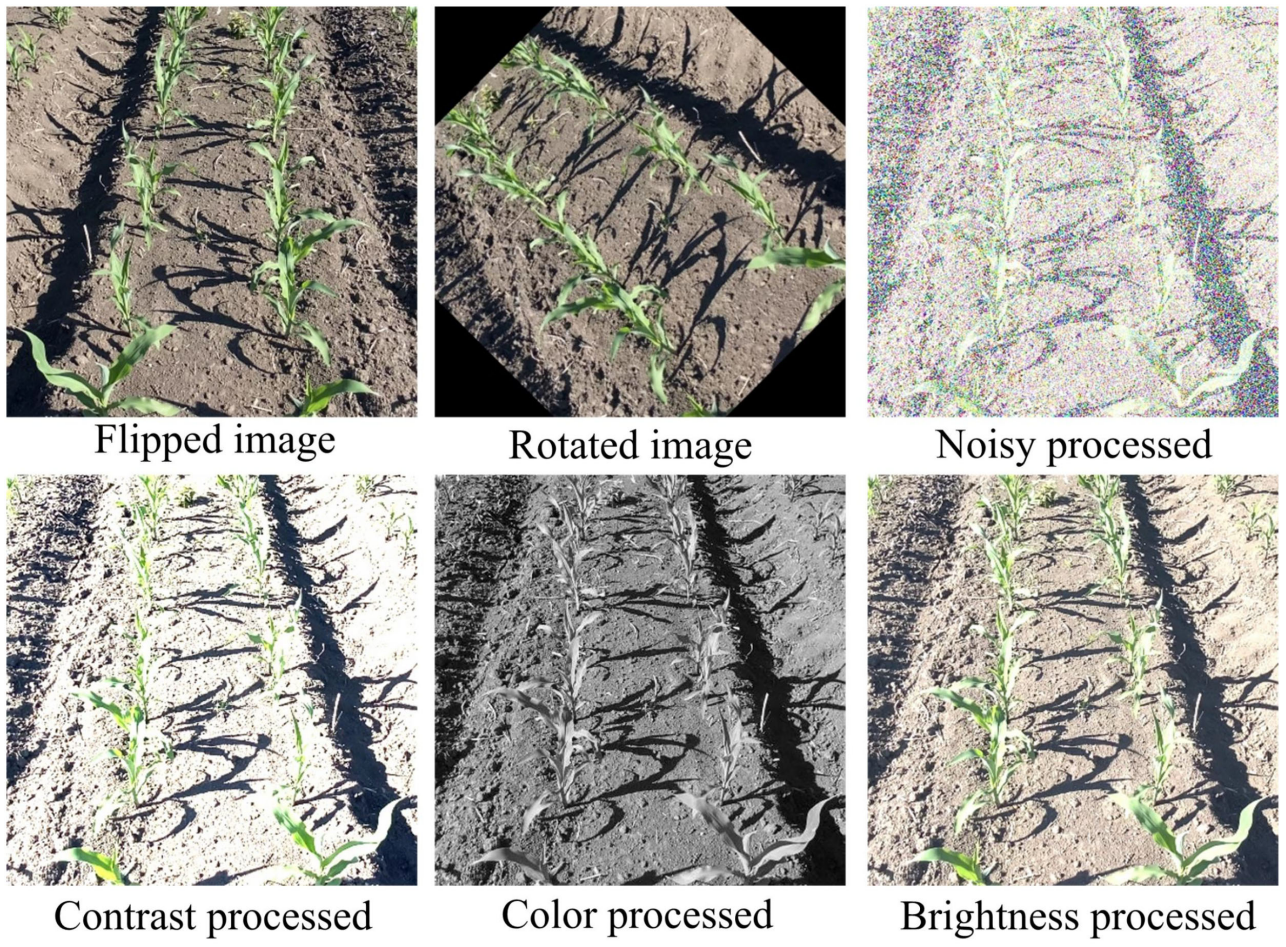
Data enhancement processing.

The original images were processed for color, brightness, contrast, noise, rotation, and mirroring as shown in **Figure 3**. Following these processes, the dataset expanded from the initial 800 to 5600 images. The augmented dataset was divided into training, validation, and test sets in an 8:1:1 ratio.

#### 2.2.2 Dataset labeling

**Figure 4:**
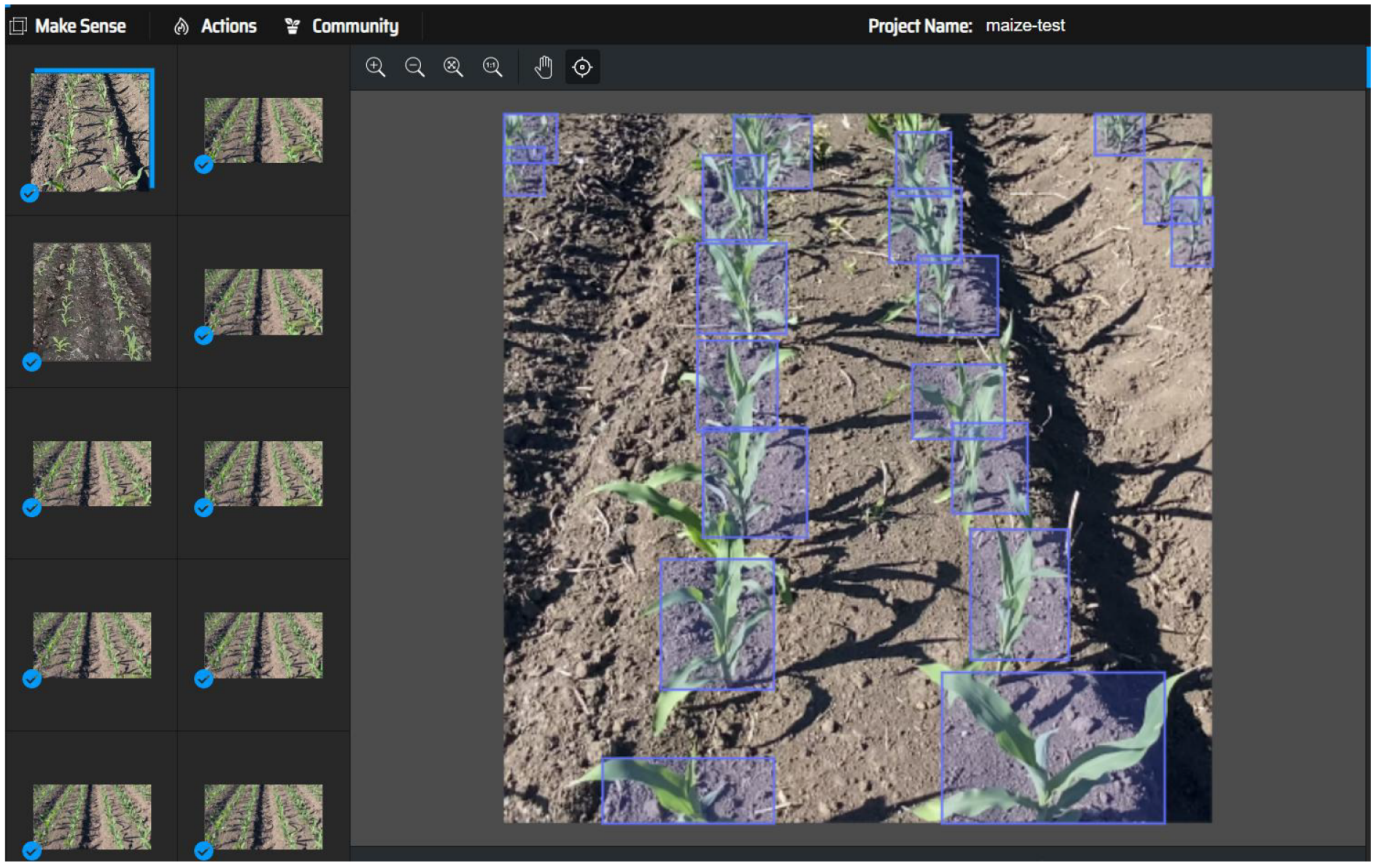
Data labeling diagram.

Prior to preparing the training dataset, meticulous labeling of the maize crop images was performed, as demonstrated in **Figure 4**. To construct a high-quality maize seedling dataset, we employed the professional image annotation software makesense, creating precise image labels through manual operation. During the data labeling phase, maize seedlings were specifically marked, allowing the you only look once object (YOLO) detection model to be optimized to recognize a single target category—maize seedlings. This single-target detection strategy significantly enhances the model’s processing speed, as computational resources are focused on identifying the sole target type rather than being dispersed among multiple categories. This approach is particularly beneficial for real-time scenarios and resource-constrained embedded systems. During labeling, special care was taken to avoid marking positive samples with unclear pixel areas. This strategy helps reduce model overfitting, where the model performs well on training data but poorly on unseen new data.

### 2.3 Methods

The primary objective of this investigation is to enhance the YOLOX-Tiny model through the development of a real-time, high-precision target detection algorithm. This advancement aims to facilitate the automatic navigation control of weeding machinery within maize fields, a critical factor in boosting weeding efficiency and diminishing the dependence on manual labor. The specific goals of our research are outlined as follows:

1. To introduce a methodology based on deep learning, capable of achieving high-precision in the recognition of maize crops under a variety of lighting conditions. This aspect is particularly vital for nocturnal operations.
2. To refine the algorithm to ensure its capability for real-time execution on embedded systems, thus meeting the online control prerequisites.
3. To enhance the accuracy of recognition, enabling the precise identification of maize within complex environmental settings.
4. To create a navigational centerline that provides accurate directional guidance for the weeding trajectory, thereby facilitating the automation of the weeding process.

YOLOX-Tiny network model, a lightweight version of YOLOX, strikes a good balance between detection speed and accuracy with its streamlined network architecture and fast detection speed (Zheng et al., 2021). This algorithm shows potential for application in field-based intelligent weeding robots (Liu et al., 2023). Therefore, in this study, considering the model size, detection accuracy, and detection speed, we have made improvements to the detection network for maize crop rows based on the lightweight YOLOX-Tiny model.

#### 2.3.1 Enhanced network architecture

**Figure 5:**
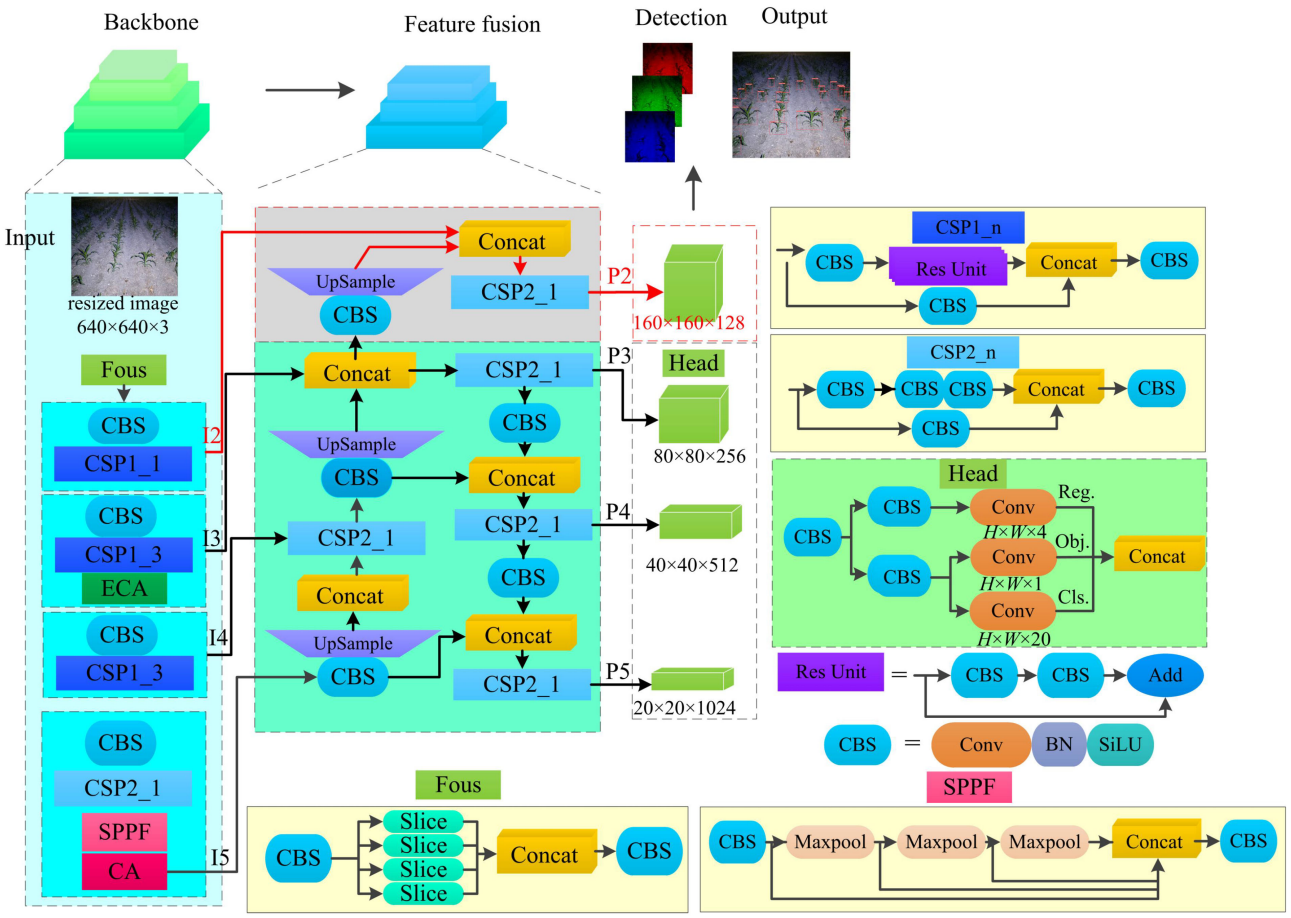
The improved YOLOX-Tiny model structure. CBS is a combination of convolution、batch normalization layer and Swish. ECA is efficient channel attention. CA is Coordinate Attention. SPPF is spatial pyramid pooling fast.

The improved network structure based on the YOLOX-Tiny network model framework is illustrated in (**Figure 5**). The highlighted sections in the figure represent the main improvements, which include multi-scale prediction and the coordinate attention module. In this structure, “Conv” denotes the depth convolution operation, “BN” represents batch normalization, “upsample” refers to the upsampling operation using the nearest neighbor algorithm, and “Concat” signifies the concatenation of feature maps. The “CBS” module consists of Conv, BN, and SiLU (Sigmoid Linear Unit) activation function.

The backbone of the network adopts the Modified CSPNet and SiLU activation function to extract deep semantic information from the input image. The “Focus” layer is composed of slice operations and basic convolution, which achieves downsampling while ensuring low parameter count and computational complexity. The spatial pyramid pooling fusion (SPPF) layer consists of max pooling operations with kernel sizes of 5×5, 9×9, and 13×13, along with basic convolution. This layer is used to enlarge the receptive field without significantly increasing the model size.

In summary, the modified YOLOX-Tiny network model incorporates improvements such as multi-scale prediction and the coordinate attention module. The backbone network employs the Modified CSPNet and SiLU activation function to extract deep semantic information. The network structure achieves downsampling through the “Focus” layer and enlarges the receptive field using the SPPF layer, while maintaining a low parameter count and computational complexity.

To enable multi-scale prediction, the YOLOX-Tiny framework incorporates the concept of the Feature Pyramid Network (FPN) and introduces an additional prediction feature map, P2. To enhance detection performance across different object scales, a specific implementation is adopted in the YOLOX-Tiny framework. Firstly, the P3 layer, which contains contextual information fused effectively, is selected. Subsequently, convolution and upsampling operations are employed to increase the size of the feature map. This enlarged feature map is then concatenated with the I2 layer, which contains detailed information. Finally, the feature map P2 is generated by fully integrating the features using the basic building block CSP2_1. By introducing this additional feature map, the YOLOX-Tiny model effectively improves the likelihood of object detection, surpassing the three prediction feature maps of the original YOLOX-Tiny model.

First, the input image is resized to 640 pixels×640 pixels. The Backbone network is then applied, producing feature maps. the predicted feature maps P2-P5 (with sizes of 160×160, 80×80, 40×40, and 20×20) are passed through a shared convolutional layer and two additional branch convolutions to decouple the localization and classification tasks. The branch convolutions consist of deformable convolution and depth convolution units (CBS). Each task-specific convolution generates predictions for the object’s position, class, and IoU-aware classification scores.

**Figure 6:**
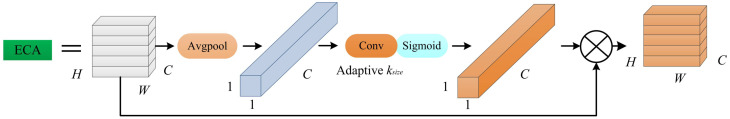
Efficient channel attention block structure.


In our study, we incorporate efficient channel attention (ECA) modules prior to CSP1-3 in the backbone network, as well as coordinate attention (CA) modules following the third CSP2-1. The ECA module is a lightweight channel attention module, as depicted (**Figure 6**). It computes channel-wise statistics of the input feature map by employing average pooling. These statistics are then multiplied with the original channels after being processed by an adaptive convolution layer and a sigmoid activation function. This multiplication operation amplifies the contribution of channels with higher importance and weakens the contribution of channels with lower importance. The size of the adaptive convolution kernel is determined using Equation 1.

(1) $$k_{size}={\left|\frac{\log_{2}C_{in}+1}{2}\right|}_{odd}$$

In the Equation 1, the variable $k_{size}$ denotes the size of the adaptive convolution kernel. The variable $C_{in}$ denotes the number of channels present in the input feature. The ${\left|\right|}_{odd}$ is used to indicate that the nearest odd number is selected.

The CA module is a lightweight channel and spatial attention module. It leverages the correlations within the given data to highlight important features. The CA module incorporates positional information into channel attention, enabling modeling of both inter-channel relationships and inter-dependencies among positional information. Furthermore, this module is plug-and-play, requiring minimal computational and parameter overhead, making it highly suitable for deployment in lightweight algorithms.

**Figure 7:**
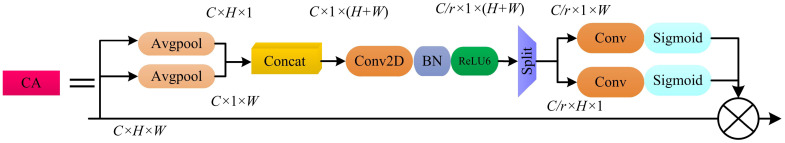
Coordinate attention block structure.

The structure of the CA module is illustrated (**Figure 7**). Given an input feature map, denoted as $I\in R^{C\times H\times W}$ , the CA module operates as follows: Firstly, two average pooling layers are applied along different spatial dimensions with pooling kernel sizes of (1, *W*) and (*H*, 1), respectively. This yields two feature vectors that aggregate information from different dimensions. This approach captures dependencies in the current spatial direction while preserving precise positional information from the other spatial direction. Secondly, the two feature vectors are concatenated through dimension transformation and then processed by a 1×1 convolution. This effectively utilizes the captured positional and correlation information to accurately highlight regions of interest. The resulting feature vector has a size of *C*/*r* × 1 × (*H* + *W*), where the parameter r controls the reduction ratio of the number of channels, enabling reduced computation and inference time. Thirdly, the feature vector is split along the spatial dimension into two independent feature vectors, which are transformed into feature maps with the same number of channels through 1×1 convolution and activation functions. Lastly, the resulting feature maps are element-wise multiplied with the input feature map.

The YOLOX-Tiny framework incorporates the ECA module and CA module in the backbone network to enhance feature representation. The enhanced features are fused with features at different scales, and the resulting feature maps are used for object detection tasks. The decoupled convolutional branches enable the network to generate predictions for object localization, classification, and IoU-aware classification scores.

The spatial pyramid pooling fast (SPPF) module is introduced at the end of the backbone network. The SPPF structure inherits the advantages of spatial pyramid pooling (SPP) and achieves the fusion of local and global features. This approach enhances the expressive power of the feature maps and facilitates the detection of objects of different sizes in the image. Moreover, the SPPF module offers faster computation speed.

#### 2.3.2 Optimization function

The maize weeding crop row recognition system requires the capability to accurately detect and locate maize crops under diverse environmental conditions. For instance, the appearance and shape of may vary with different weather conditions, such as under intense sunlight or during overcast, rainy weather. By incorporating an augmented loss function, the model can be rendered more robust, enabling it to better adapt to these variations and accurately detect and locate maize seedlings.

During the training phase for crop object detection tasks, it was observed that the target localization loss converges slowly, especially for small objects in the image. Due to the small size of the objects, the predicted bounding boxes often fall within the ground truth boxes, resulting in an inclusion relationship between them. The complete intersection over union(CIoU) loss function improves the accuracy of predicted bounding boxes, enabling them to be aligned with the positions of the ground truth boxes.

When the intersection over union(IoU) loss function is employed as the regression loss for bounding boxes, it fails to provide accurate displacement directions for the bounding boxes in the presence of overlap issues. Consequently, this problem leads to subpar performance in image localization. The CIoU loss function is an improved version of the IoU loss function, which incorporates the calculation of center position and aspect ratio errors. On one hand, it addresses the issue of overlapping bounding boxes. On the other hand, it takes into account the overlap area, distance between center points, and aspect ratio, resulting in faster convergence of the loss function. Therefore, in this study, the CIoU loss function is adopted as the formula for bounding box regression loss, as shown in Equation 2.


(2) $$\left\{ \begin{array}{l} L_{\text{CIoU}}=1-R_{\text{IoU}}+\frac{\rho (b,b^{gt})}{c^{2}}+\alpha v\\ \alpha =\frac{v}{(1-R_{\text{IoU}})+v},\\ v=\frac{4}{\pi^{2}}(\text{arctan}\frac{w^{gt}}{h^{gt}}-\text{arctan}\frac{w}{h}{)}^{2} \end{array}\right.$$

In the Equation 2, the term $R_{\text{IoU}}$ denotes the intersection over union between the predicted and ground truth bounding boxes. The variable ρ denotes the Euclidean distance between two points. The variables b and $b^{gt}$ denote the center point of the predicted and ground truth bounding boxes, respectively. The variable *c* denotes the diagonal distance between the minimum bounding boxes of the predicted and ground truth bounding boxes. The parameter *α* is used to adjust the coordination ratio between the predicted and ground truth bounding boxes. The parameter *v* is used to measure the aspect ratio consistency between the bounding boxes. Finally, the variables $w^{gt}$ , w, $h^{gt}$ and *h* denote the width and height of the predicted and ground truth bounding boxes.

The IoU-aware classification loss and category loss are computed using the cross-entropy loss function. In the later stages of model training, an additional $L_{1}$ regularization boundary box loss function is incorporated to further reduce the model’s localization error. Therefore, this study adopts the CIoU loss function as the bounding box regression loss function, as shown in Equation 3.


(3) L=1NposCrossEntropy(PCls.pos,GCls.pos)+λ1NposCrossEntropy(PObj.pos+neg,GObj.pos+neg)+λ2NposLIoU(Pboxpos,Gboxpos)+λ3NposL1(PReg.pos,GReg.pos)

In the Equation 3, the term *CrossEntropy* denotes the loss function known as cross-entropy. The $L_{IOU}$ term denotes the loss function called CIoU. The $L_{1}$ term denotes the regularization boundary box loss function. $N_{pos}$ denotes the count of positive samples. The variables *P* and *G* denotes the predicted and ground truth samples, respectively. The terms *pos* and *neg* denotes the positive and negative training samples, respectively. *Cls*., *Obj*., *box*, and *Reg.* denotes the classification scores, IoU-aware scores, bounding boxes, and localization offsets, respectively.

#### 2.3.3 Image enhancement preprocessing

**Figure 8:**
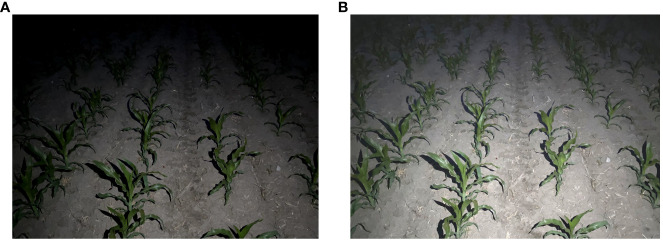
Differences in images under different distances from the light source. **(A)** Low-light image; **(B)** High-light image.

To enable all-weather operations, a floodlight point light source was utilized for active illumination during nighttime maize crop image acquisition (Chen and Chiu, 2023; Jia et al., 2023). However, the nighttime illumination method falls short of replicating the illumination effect of parallel sunlight during the daytime, resulting in a certain degree of degradation in the captured maize crop images (Rahman et al., 2016; Liu et al., 2018; Liao et al., 2020). These challenges include a low overall illumination level in the field of view and limited color contrast between the maize plants and the background, especially at the image edges. These factors can significantly hinder the effectiveness of subsequent machine vision recognition tasks (Zhang et al., 2024). As shown in **Figure 5**, the significant differences in image characteristics resulting from varying light intensities are evident. The low-light maize image in **Figure 8A** exhibits texture and color degradation, leading to reduced contrast between the foreground and background. In contrast, the high-light maize image in **Figure 8B** is bright and clear, facilitating target detection.

To facilitate recognition under various lighting conditions, our approach incorporates a Gamma image enhancement algorithm for image preprocessing, inspired by the Retinex theory. This algorithm is designed to accentuate crop contours and details, thereby enhancing the recognition capability of images captured under low-light conditions. We collected images under different lighting conditions, observing that images from areas closer to the light source exhibited higher brightness and were easier for target detection, whereas images from areas further from the light source suffered from reduced quality and diminished foreground-background contrast. The Gamma image enhancement algorithm employs an adaptive adjustment of image brightness by calculating the number of pixels at each brightness level. By integrating the enhanced brightness layers, the output image achieves a more uniform brightness. Such preprocessing effectively mitigates the issue of image quality variation due to different distances from the light source, thus improving the model’s recognition performance across various lighting conditions.

The main steps of this image enhancement algorithm are as follows:

Separating the brightness component and reflection component of the image using the Retinex theory, as shown in Equation 4.


(4) $$R^{c}(x,y)=\frac{I^{c}(x,y)}{L(x,y)},c\in \{r,g,b\}$$

In the Equation 4, denotes the separated reflection component, $I^{c}(x,y)$ denotes the brightness of each channel in RGB, and L(x,y) denotes the brightness component of the image.

The adaptive gamma correction algorith $R^{c}(x,y)$ m is utilized to correct the brightness component. $L_{en}(x,y)$ represents the corrected luminance component, as shown in Equation (5).


(5) $$L_{en}(x,y)=L{\left(x,y\right)}^{\gamma (x,y)}$$

Where γ(x,y) denotes the coefficient matrix.

Determine the parameters for Gamma correction based on the distribution of brightness values, as shown in Equation 6.


(6) $$\gamma (l)=1-\sum_{v=0}^{l}\frac{P_{w}(v)}{s_{p}}$$

(7) $$s_{p}=\sum_{v=0}^{l}P_{w}(l)$$

In the Equation 7, $\sum_{v=0}^{l}\frac{P_{w}(v)}{s_{p}}$ denotes the cumulative distribution function of the brightness component, and $P_{w}(l)$ denotes the weight distribution function of each brightness value. $P_{w}(l)$ represents the weight distribution function of each brightness value, as shown in Equation 8.


(8) $$P_{w}(l)=\frac{P(l)-P_{\min}}{P_{\max}-P_{\min}}$$

In the Equation 8, P(l) denotes the probability density function of the brightness component


(9) $$P(l)=\frac{n_{l}}{n_{p}}$$

In the Equation 9, $n_{l}$ denotes the number of pixels corresponding to the respective brightness, and $n_{p}$ denotes the total number of pixels in the brightness component.

The final enhanced image, $L_{en}^{c}(x,y)$ is obtained by fusing $L_{en}(x,y)$ and $L_{en}(x,y)$ , thereby restoring the original image’s color and details, as shown in Equation 10.


(10) $$L_{en}^{c}(x,y)=R^{c}(x,y)\cdot L_{en}(x,y)$$

**Figure 9:**
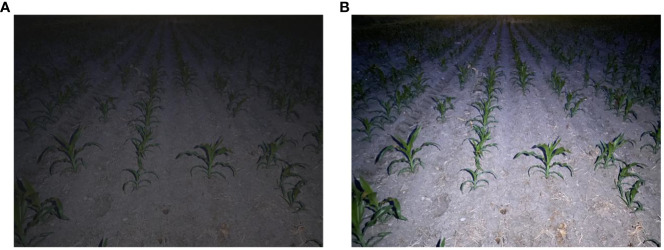
Variation in image brightness. **(A)** Original night low illumination image; **(B)** Gamma transformed image.

**Figure 10:**
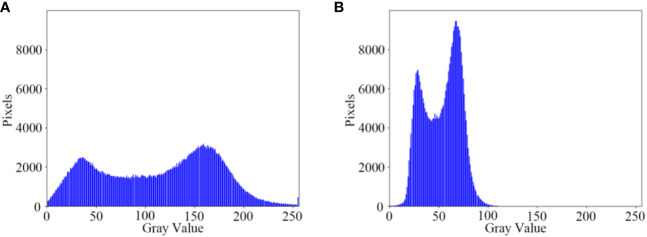
Comparison of image grayscale. **(A)** Original night low illumination image Gamma; **(B)** Gamma transformed image.

The brightness enhancement effect of the illumination adjustment module on low-light nighttime images is demonstrated (**Figure 9**). The low-light images undergo Gamma transformation, resulting in an overall increase in brightness in the image field. This enhancement improves the distinction between foreground targets and background images, thereby facilitating the detection of maize crop targets at long distances. Additionally, the grayscale variation of the low-light maize crop rows before and after brightness enhancement by the illumination adjustment module is depicted (**Figure 10**). After the illumination adjustment, the grayscale distribution of the image becomes more uniform, with a larger number of pixels distributed in the middle range of intensities. This improvement in visual quality and accurate identification of maize crops contributes to enhancing the overall performance.

#### 2.3.4 Computation of the central navigation line for crop row fitting

**Figure 11:**
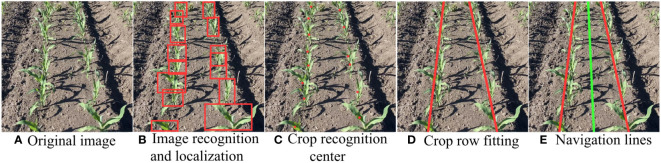
Fitting of Crop Row Navigation Lines. **(A)** Original image; **(B)** lmage recognition and localization; **(C)** Crop recognition; **(D)** Crop row fitting; **(E)** Navigation lines.

(1) During the identification process, maize plants are also present around the periphery of the captured image. Thus, to accurately extract target position information, a predefined crop row area is established, which allows for the extraction of targets solely within the set boundaries. The target area encompasses the left and right crop rows, which can be approximately delineated using image processing techniques, as illustrated in **Figure 11A**.
(2) Acquisition of target position information is achieved through the refined output of the YOLOX-Tiny model, which provides bounding box information of the detected targets as shown in **Figure 11B**. This includes the coordinates of the top-left and bottom-right corners of the bounding box. The central coordinates of the targets are computed by determining the horizontal and vertical midpoints of the bounding boxes, respectively. Consequently, this establishes datasets for the coordinates of the left and right crop rows. The central coordinates of the targets serve as the independent variable,ith the corresponding y -coordinates as the dependent variable. Datasets are formed by pairing all central x -coordinates with their corresponding y -coordinates.

In steps (1) and (2), beyond setting the crop row boundaries, additional detection logic can be incorporated to identify instances of missing plants and offsets in seedling positioning. For instance, missing plants can be detected by calculating the distance between successive plant positions. If the distance exceeds a predefined threshold, it can be flagged as a potential gap in planting. A distance threshold $\left(D_{thresh}\right)$ is established, and for each seedling pair *i* and *i*+1, the vertical distance ( ${\text{D}}_{i,i+1}$ ) is calculated. If $\left(D_{i,i+1}>D_{thresh}\right)$ , the point *i*+1 is marked as the starting point of a missing plant. The recognition results of missing plants and offsets are then used to refine the dataset. Points marked as gaps can be selectively ignored.

(3) To mitigate the influence of off-center seedlings on the fitting results, a weighted least squares method is employed. The fundamental principle of the least squares method is to determine the parameters of the fitting line by minimizing the sum of squared residuals. The weighted least squares fitting is applied to the dataset to obtain the line parameters. For each data point $\left(x_{i},y_{i}\right)$ , a weight $w_{i}$ is assigned based on its distance from the center of the crop row, with points closer to the center receiving higher weights.

The objective function of the weighted least squares method aims to minimize the sum of the weighted squared residuals, as shown in Equation 11:


(11) $$S=\sum_{i=1}^{n}w_{i}{\left(y_{i}-\left(mx_{i}+b\right)\right)}^{2}$$

In the Equation 11, m denotes the slope, b denotes the intercept, and $w_{i}$ denotes the weight for the *i*th data point.

Solving the weighted least squares problem typically involves resolving the weighted normal equations, as shown in Equation 12:


(12) $$A=\left[\sum w_{i}x_{i}^{2}\;\sum w_{i}x_{i}\;\sum w_{i}x_{i}\;\sum w_{i}\right]\left[\begin{array}{cc} m & b \end{array}\right]\left[\sum w_{i}x_{i}y_{i}\;\sum w_{i}y_{i}\right]$$

By solving this set of equations, we obtain the parameters m and b for the weighted least squares fit.

The equation for the central navigation line is presented in Equation 13. With the equations of the two fitted crop row lines $y=m_{1}x+b_{1}$ and $y=m_{2}x+b_{2}$ , as shown by the two red lines in **Figure 11C**, the intersection point (if they intersect) of these two lines is computed by solving the system of equations to find the point (*x_i_*, *y_i_*). The central navigation line is the line that connects the midpoint of the two lines.


(13) $$m_{1}x+b_{1}=m_{2}x+b_{2}$$

The slope of the central line (*m_c_*) is computed as the harmonic mean of the slopes of the two lines, as depicted in Equation 14:


(14) $$m_{c}=\tan(\frac{\arctan(m_{1})+\arctan(m_{2})}{2})$$

The intercept (*b_c_*) denotes determined by substituting the intersection point (*x_i_*, *y_i_*) into the central line’s Equation 15:


(15) $$y=m_{c}x+b_{c}$$

For efficient computation, matrix operation libraries are utilized to perform the least squares calculations, leveraging optimized linear algebra operations for enhanced speed. The fitting result, depicted in **Figure 11D**, produces the green central navigation line. This line guides the weeding machinery along the navigational path, preventing damage to the crops. In practical applications, particularly in machine vision and autonomous navigation systems, this central line serves as an estimation to direct robots or equipment navigation between two crop rows.

### 2.4 Experimental methodology

#### 2.4.1 Model training

The experimental hardware platform was a dell workstation equipped with an intel core i7-12700H processor, 32GB of RAM, and an nvidia GeForce RTX 4070 graphics card. The operating system was Windows 11, with the deep learning framework being PyTorch version 2.0.1, and Python 3.9.

The experiment employed uniform training parameters as outlined in **Table 1**. The optimizer used was standard stochastic gradient descent (SGD), with momentum, decay coefficient, and Nesterov momentum set to 0.9, 0.0005, and True, respectively. These settings were chosen to facilitate accelerated convergence and enhance the generalization capability of the model. A cosine annealing learning rate strategy was implemented as the learning rate scheduler, balancing rapid convergence with avoidance of local optima. The minimum learning rate was set to 5% of the initial rate and was employed during the final 15 epochs to maintain model stability in the latter stages of training. The batch size was set to 16, with a total of 400 epochs.

**Table 1:** Training parameters.

Name	Value
Optimizer	SGD
Momentum	0. 9
Weight decay	5×10^−4^
Nesterov	True
Learning rate scheduler	Type is CosineAnealing, Learning rate is 0. 0025, Min lr ratio is 0. 05
Batch	16
Epoch	400
Mosaic	Img scale is (640, 640)

For the training set preprocessing, a mosaic data augmentation strategy was adopted. This strategy enhances the model’s generalization ability and detection accuracy by combining multiple images and a variety of augmentation operations to simulate a richer set of training samples. The online Mosaic augmentation method, which randomly applies image enhancement transformations during training, is widely used in deep convolutional model training to bolster detection precision and generalization performance. However, given that images augmented with the Mosaic technique can deviate from natural appearances, this method was utilized only for the first 280 epochs. Subsequently, the mosaic augmentation was disabled to prevent overfitting, thus reinforcing the model’s learning of complex scenarios and targets in the initial training phase while avoiding excessive fitting in the later stages.

#### 2.4.2 Evaluation metrics

This study assesses the effectiveness of the detection model through various metrics, including precision ( P ), recall ( R ), $F_{1}-\text{score}$ , average precision ( AP ), and Detection Speed (Carvalho et al., 2019; Steurer et al., 2021). Detection Speed refers to the time required by the model to process a single image. The samples can be categorized into four types based on the combination of their actual class and the predicted class by the model: True positive ( ${\text{T}}_{\text{P}}$ ), where the sample is positive and predicted as positive; False negative ( ${\text{F}}_{\text{N}}$ ), where the sample is positive but predicted as negative; False positive ( ${\text{F}}_{\text{P}}$ ), where the sample is negative but predicted as positive.

Precision ( P ): Precision quantifies the proportion of positive instances correctly identified by the model out of all the positive instances it detects. This metric evaluates the model’s accuracy in identifying true positives, effectively measuring the extent of false positive errors. The precision is shown in Equation 16:


(16) $$P=\frac{T_{P}}{T_{P}+F_{P}}\times 100\%$$

In the Equation 16, True positive ( $T_{P}$ ) denotes the number of correctly detected maize instances, and False negative ( $F_{P}$ ) represents the number of times the model erroneously identifies the background as maize.

Recall ( R ): the proportion of positive instances that the model successfully detects out of the total actual positive instances. It assesses the model’s ability to recognize true positives, gauging the extent of false negative errors. The recall is shown in Equation 17:


(17) $$R=\frac{T_{P}}{T_{P}+F_{N}}\times 100\%$$

In the Equation 17, True negative ( $F_{N}$ ) denotes the number of maize instances that the model fails to detect.

Average precision ( AP ): the model’s accuracy in localizing and classifying targets within images. A higher AP value signifies superior target detection performance, indicating the model’s proficiency in accurately identifying and localizing targets with minimal false positives and negatives. The AP is shown in Equation 18:


(18) $$AP=\int_{0}^{1}P(R)dR$$

In the Equation 18, P(R) denotes the integrated area under the precision-recall curve for a single-class detection target. $F_{1}-\text{score}$ : the harmonic mean of precision and recall, providing a composite measure of the model’s precision and recall. A higher $F_{1}-\text{score}$ denotes a more optimal balance between precision and recall. The $F_{1}-\text{score}$ is shown in Equation 19:


(19) $$F_{1}=\frac{2PR}{P+R}\times 100\%$$

#### 2.4.3 Assessment of fit line evaluation

To evaluate whether the crop row fit lines meet the established criteria, the average fit time and average angular error are used as assessment metrics.

The difference in radians between manually annotated and algorithmically fitted crop row lines is compared to calculate the angular difference between the two lines, as shown in Equation 20:


(20) $$\Delta \theta =\left|\arctan(a_{1})-\arctan(a_{2})\right|$$

In the Equation 20, $a_{1}$ and $a_{2}$ are the slopes of the two lines, respectively. A smaller angular difference indicates a minor angular deviation between the two lines.

The average fit time for crop rows is calculated, as shown in Equation 21:


(21) $$t=\frac{\sum_{m=1}^{M}t_{m}}{M}$$

In the Equation 21, $t_{m}$ denotes the fitting time required for the crop rows in image m , and M denotes the total number of images.

The average angular error for crop rows, $n_{a}$ denotes calculated as presented in Equation 22:


(22) $$n_{a}=\frac{1}{A}\sum_{i=1}^{A}\Delta \theta_{i}$$

In the Equation 22, A denotes the total number of maize crop rows, and $\Delta \theta_{i}$ denotes the fitting deviation for the *i*-th row.

## 3 Results

This chapter delves into a thorough analysis and discussion of the experimental results obtained from the enhanced YOLOX model in maize crop recognition tasks, juxtaposing these findings with other pertinent studies. Initially, Section 3.1 provides a detailed evaluation of the performance of the improved YOLOX-Tiny model on the test dataset, including key metrics such as recognition accuracy and false detection rates under various lighting conditions and scenarios. Subsequently, Section 3.2 employs feature visualization techniques to observe the features extracted by the model at different convolutional layers, thereby analyzing the enhancement in the model’s recognition capabilities. Section 3.3 presents the experimental outcomes of our proposed least squares method for fitting navigation routes, including metrics such as fitting time and angular error.

### 3.1 Model training results and analysis

To comprehensively evaluate the effectiveness of the proposed improvements in this study, we conducted a series of cumulative experiments with the enhanced algorithm and summarized the results in **Table 2**. By comparing the data in the table, we can gain a detailed understanding of the performance and related parameter configurations of different models in the task of object detection.

**Table 2:** Performance comparison of algorithm Enhancements in a cascade manner.

Models	Average precision *AP*/%	Parameters/10^6^	Detection time/ms
YOLOX-Tiny+LA	90.21	5.04	11.40
+multi-scale prediction method	92.42	5.40	14.3
+Efficient Channel Attention, ECA+coordinate attention CA	92.15	5.41	15.6
+loss function (The improved YOLOX-Tiny model)	92.17	5.6	15.6

Initially, the YOLOX-Tiny model, augmented with the illumination adjustment module, achieved an average precision of 90.21%, with a model parameter count of $5.04\times 10^{6}$ , and a detection time of 11.40 milliseconds. These results indicate that the model demonstrates commendable performance in object detection tasks, accurately identifying and localizing target objects.

Further, researchers improved the model by adopting a multi-scale prediction approach, which increased the AP to 92.42%. Multi-scale prediction enables the model to detect targets at various scales, effectively adapting to and recognizing objects of different sizes, thereby significantly enhancing detection accuracy.

Moreover, this study also incorporated mechanisms such as efficient channel attention and coordinate attention. Although the introduction of these attention mechanisms resulted in a slight decrease in AP to 92.15%, they aid the model in focusing more intently on the key features of targets, thus improving the precision and robustness of detection.

Finally, we optimized the loss function to further refine the YOLOX-Tiny model, elevating the AP to 92.2%. This outcome suggests that the optimized loss function can more effectively guide the model in learning the feature representations of target objects, thereby improving detection performance. Although enhancements in model performance were accompanied by an increase in the number of parameters, the detection time remained relatively stable. Overall, by implementing multiple optimizations on the model, we have not only significantly improved model performance but also maintained a low detection time, achieving a high AP value.

**Figure 12:**
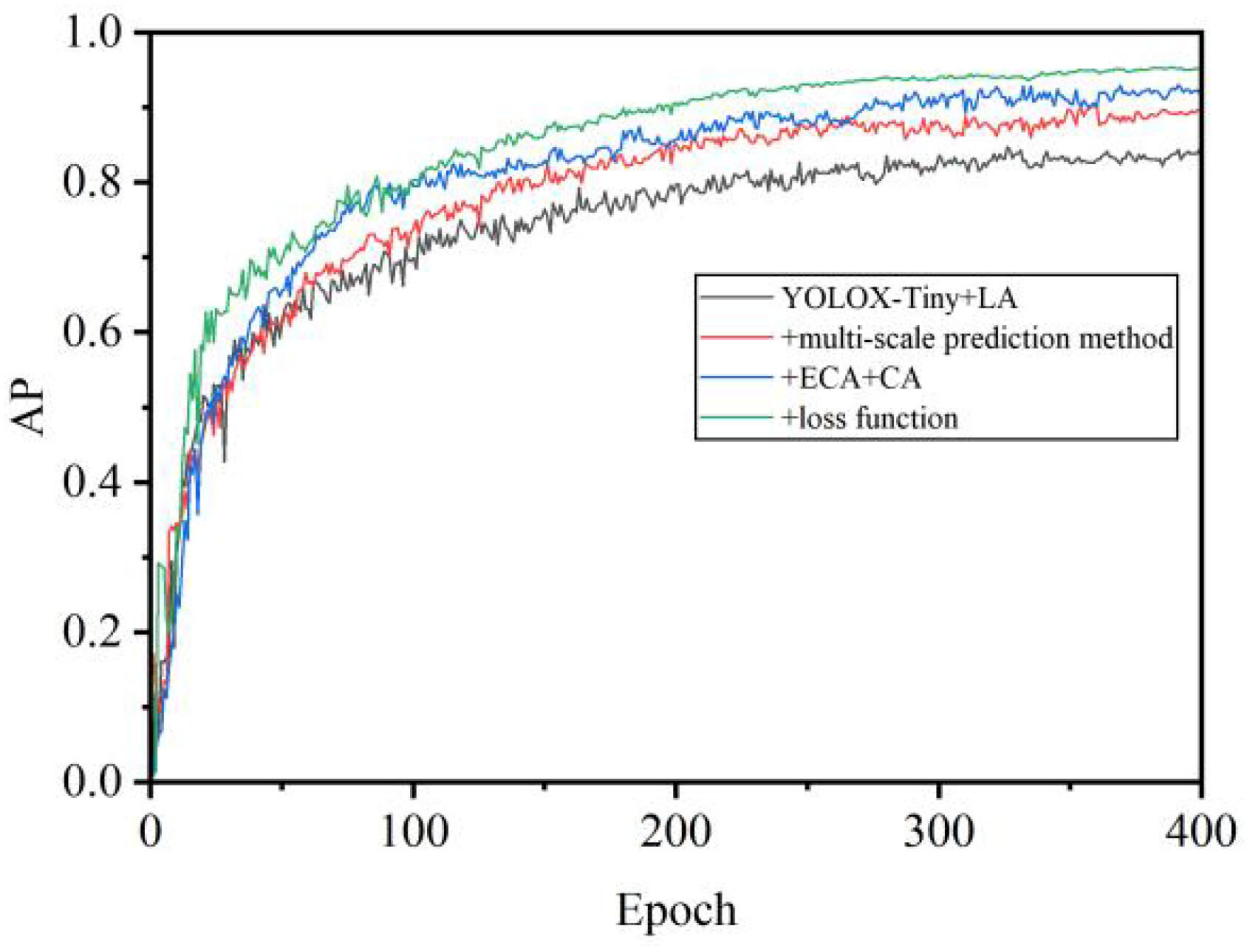
AP curve during training.

**Figure 13:**
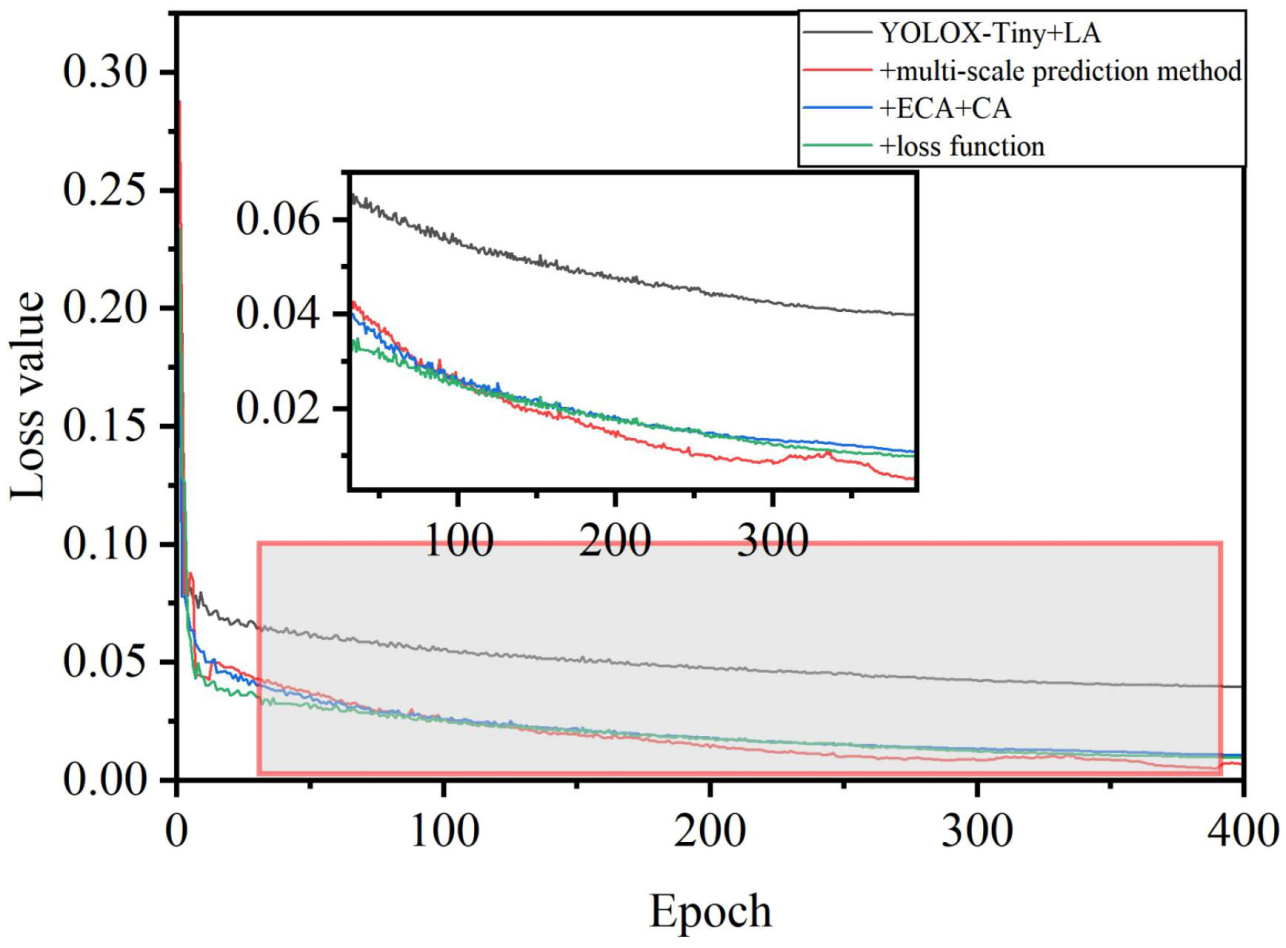
Loss curve during training.

The evolution of Average Precision and the loss function during the training process of the model was depicted in **Figures 12** and **13**. Initially, there was a significant improvement in accuracy, while the loss function showed fluctuations. After 100 epochs, both accuracy and the loss function stabilized, indicating the model’s transition to the fine-tuning phase. This transition was attributed to the exclusion of Mosaic data augmentation, which addressed inaccuracies in object annotations caused by excessive augmentation. Furthermore, adopting a fixed minimum learning rate strategy addressed concerns regarding sluggish weight updates. Additionally, incorporating the L1 regularization bounding box loss function effectively reduced object localization errors and improved sensitivity to localization deviations. As the algorithm underwent iterative enhancements, there was an observable acceleration in convergence speed and an improvement in recognition accuracy. The refined network architecture notably amplified accuracy, while the numerical disparities in the loss function were relatively insignificant. However, enhancing the loss function resulted in a noticeable decrease in its numerical value, accompanied by corresponding accuracy gains.

To evaluate the accuracy, robustness, and stability of the improved YOLOX-Tiny model, we trained four other object detection networks on the same dataset: SSD, YOLOV4-Tiny, YOLOv5s, and YOLOX-Tiny. The SSD model used images with dimensions of 512×512 pixels, while the other models utilized images with dimensions of 640×640 pixels for testing the model’s detection time. The results are presented in **Table 3**.

**Table 3:** Detection performance comparison of five models.

Models	Average precision *AP*/%	*F*_1_ score/%	Recall *R*/%	Precision *P*/%	Detection time/ms	Model size/MB
SSD	89.2	84.5	78.2	93.4	20.7	91.2
YOLOv4-Tiny	89.6	85.6	86.4	88.4	7.62	23.4
YOLOv5s	91.2	89.1	87.5	90.2	9.07	40.7
YOLOX-Tiny	90.5	88.7	88.1	91.6	13.4	20.1
the improved YOLOX-Tiny model	92.2	90.2	89.1	92.4	15.6	18.6

Based on the data and comparative analysis presented in **Table 3**, we can draw the following conclusions:

The proposed optimized model outperformed the other models in terms of maize crop row detection. It achieved the highest scores in mean average precision (92.2%), *F*_1_-score (90.2%), recall (89.1%), and precision (92.4%), indicating its high accuracy and comprehensiveness in identifying maize crop rows.

In comparison, the SSD model had lower average precision (89.2%) and *F*_1_-score (84.5%). Although it had the highest detection accuracy (93.45%), it missed approximately twice as many maize seedlings compared to the improved YOLOX-Tiny model. Additionally, the SSD model had a large model size of 95MB, making it unsuitable for embedded devices. On the other hand, the YOLOV4-Tiny model had the shortest detection time (7.62 milliseconds) but exhibited the lowest detection accuracy (89.6%). It also had a higher number of background misclassifications as crops, indicating limitations in accuracy. In contrast, the improved YOLOX-Tiny model achieved a good balance between model size, detection accuracy, and speed. It had a detection time of 15.6 milliseconds, a 16.4% improvement over the YOLOX-Tiny model, while still maintaining high detection accuracy. Furthermore, the improved YOLOX-Tiny model had a smaller model size of 18.6 MB, a 7.1% reduction compared to the YOLOX-Tiny model. These findings suggest that the improved YOLOX-Tiny model is suitable for embedded devices, offering both high accuracy and faster detection speed with a smaller model size.

In conclusion, the proposed optimized model demonstrated the best overall performance in maize crop row detection, with higher average precision, *F*_1_-score, recall, and precision. It is well-suited for deployment on embedded devices, which has significant implications for practical applications in maize crop row detection tasks.

**Figure 14:**
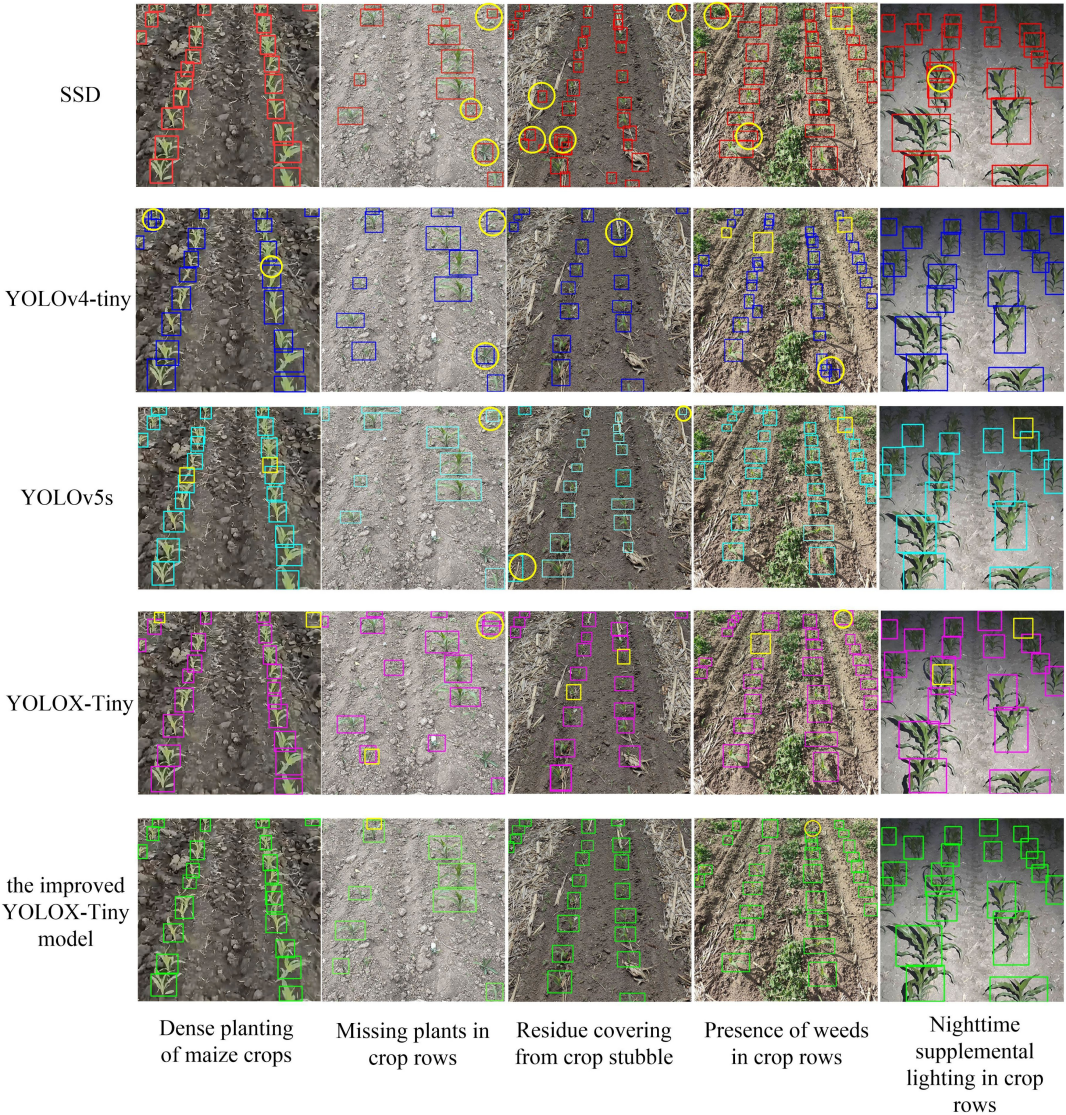
Crop detection results of 5 models. The blue boxes are the detection box for five models, the yellow boxes and circles are the manually marked missed and falsely detected boxes, respectively.

The detection results (**Figure 14**) were used to compare the performance of five models in various aspects, such as dense maize crops, missed seedlings in crop rows, straw residue coverage, presence of weeds in crop rows, and nighttime supplemental lighting. The SSD model exhibited missed detections and false detections, especially in scenarios with higher weed presence and straw residue coverage. Missed detections were more prominent in nighttime conditions, indicating the vulnerability of the SSD model to dense detection areas and environmental disturbances. The YOLOV4-Tiny model showed improved performance in detecting dense targets but struggled with accurately identifying small targets occluded by leaves and distant crops. Nighttime lighting conditions and occlusions between crops also affected the detection performance of the YOLOv4-Tiny model, leading to missed detection issues. The YOLOX-Tiny model achieved higher accuracy in detecting close-range targets but compromised recognition accuracy for small distant target crops due to its lightweight network. However, compared to the SSD and YOLOv4-Tiny models, the YOLOX-Tiny model adapted better to different lighting conditions and could detect occluded fruits and crops. It accurately identified cases where small distant target crops were recognized as a single crop due to leaf occlusion. In regions with rich weed coverage, it accurately identified crops that were occluded by leaves and mistaken as a single crop. In nighttime crop rows, it performed well in areas with close-range strong lighting but had decreased recognition accuracy in areas with insufficient distant lighting, potentially mistaking crops passing through as a single crop due to leaf occlusion.

The YOLOv5s model exhibited high overall detection accuracy but struggled with identifying small distant targets and occasionally produced false positives. In this study, the improved YOLOX-Tiny model was introduced, building upon the strengths of YOLOX-Tiny while addressing missed detections and false positives. The improved model demonstrated further advancements in recognition accuracy, particularly in low-light conditions, effectively identifying crops even in the presence of straw residue coverage. In challenging field conditions characterized by dense weed growth, the model accurately discerned crop features and achieved heightened precision in crop identification under low-light conditions. Overall, the proposed the improved YOLOX-Tiny model represented a significant enhancement in crop detection performance.

### 3.2 Model interpretability and feature visualization

**Figure 15:**
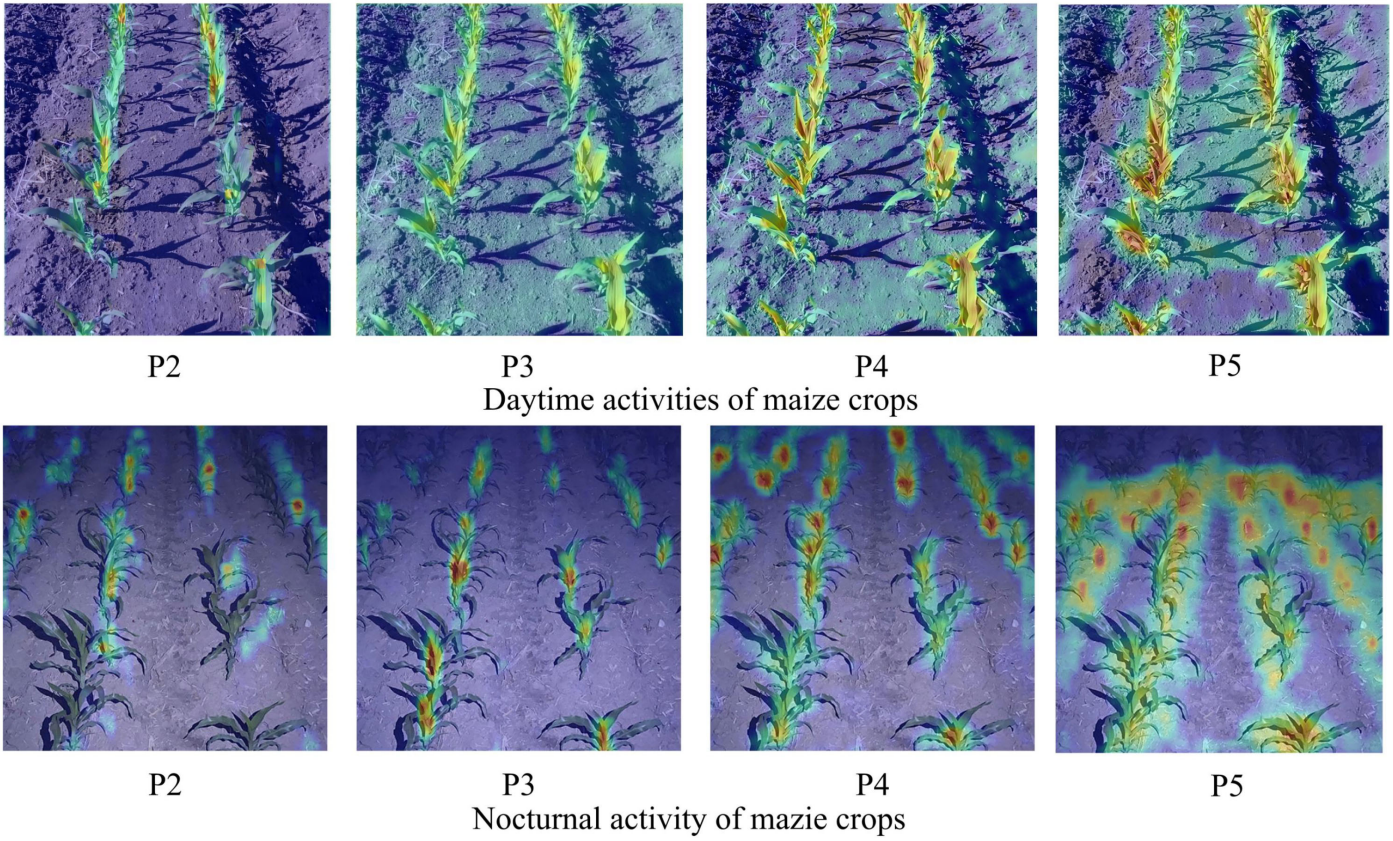
Thermal map visualization results. The redder the color, the greater the output value of the convolution layer.

The current process of object detection using convolutional neural networks (CNN) lacks adequate explanation, thereby limiting our comprehension of the learned object features and impeding further optimization of the model structure. To enhance the interpretability of the model, feature visualization techniques are employed, wherein the features extracted by different convolutional layers are transformed into visual images. These images effectively portray the distinctive features extracted by each convolutional layer. In order to facilitate a better understanding of the features extracted by convolutional neural networks, particularly before and after network structure improvements, we conducted a visualization analysis of the features from the P2-P5 multi-scale prediction layers. We specifically focused on the recognition of crops under daylight and nighttime supplemental lighting conditions, as depicted in **Figure 15**.

The improved YOLOX-Tiny model’s backbone network extracted features such as color and texture from crops and background, enabling the identification of maize crops and their corresponding background regions. As the network depth increased and features were fused, it further extracted abstract features of crops while smoothing out background information. Subsequently, all the extracted features were integrated within the detection network, effectively removing background noise and highlighting the morphology of maize crops. This study presented the extracted features of maize crop detection in complex natural environments using the improved YOLOX-Tiny model, focusing on the perspective of feature extraction and elucidating the process of maize crop detection using convolutional networks. The proposed enhancements in the algorithm resulted in superior coverage of objects of varying scales, surpassing the original YOLOX-Tiny model in terms of response intensity and boundary detection. The coordinate attention module extracted crucial channel information, effectively filtering out background noise, while the deformable convolution offset the sampling points based on the object’s shape, enabling more precise coverage of the objects.

### 3.3 Crop row centerline detection experiment

The accuracy of maize crop row recognition in this study was unaffected, even though the images within the bounding boxes were not preprocessed before locating the feature points. The proposed model also reduced the fitting time for the centerlines of the crop rows. The improved YOLOX model was deployed on a mobile device for real-time testing of its detection and fitting performance. The mobile device used was a 10-inch capacitive industrial tablet PC equipped with an Intel(R) Celeron(R) CPU J1800 processor, 2GB of RAM, and a 32-bit Windows 7 operating system. To validate this claim, we compared the fitting results obtained using the proposed least squares method combined with the center point of the bounding box with the fitting results obtained using other methods mentioned in the reference (Diao et al., 2023). These methods include the SUSAN corner detection method + least squares method (Lai et al., 2023), the Means method + least squares method (Liu et al., 2020), and the FAST corner detection method + least squares method (Montalvo et al., 2012). We calculated the radian difference between the manually labeled and fitted crop row lines, which represents the angular deviation between the two lines.

**Figure 16:**
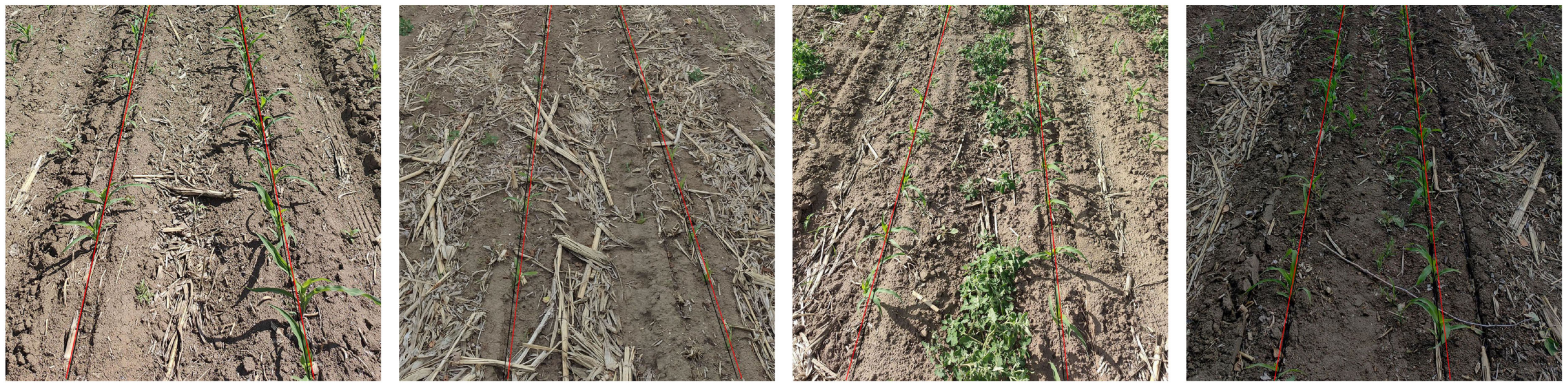
Extraction results of navigation lines in maize crop rows.

The results of extracting the navigation lines of the intelligent weeding machinery using the improved YOLOX-Tiny model (**Figure 16**). The red line indicates the annotated results obtained from the algorithm proposed in this study, while the black line represents the manually annotated baseline of the crop rows.

Based on the results evaluated in **Table 4**, the SUSAN corner detection method + least square method had a fitting time of 85.6 milliseconds and an average angle error of 0.87 degrees, indicating a longer fitting time and a larger angle error. The Means method + least square method had a fitting time of 74.8 milliseconds and an average angle error of 0.95 degrees. The FAST corner detection method + least square method had a fitting time of 67 milliseconds and the highest average angle error of 1.98 degrees. The improved YOLOX-Tiny model + least square method had the shortest fitting time of 42 milliseconds and the lowest average angle error of 0.59 degrees. This indicated that the improved YOLOX-Tiny model + least square method was both efficient and accurate in fitting centerlines of maize crop rows. Overall, the improved YOLOX-Tiny model + least square method performed the best in terms of fitting time and angle error. It was both efficient and accurate.

**Table 4:** Evaluation of Results for Centerline Fitting.

Evaluation index	SUSAN corner detection method + least square method	Means method + least square method	FAST corner detection method + least square method	the improved YOLOX-Tiny model+least square method
Average fitting time *t*/ms	85.6	74.8	67	42
Average angle error *n*/°	0.87	0.95	1.98	0.59

## 4 Discussion

In the realm of modern agriculture, intelligent agricultural machinery plays a crucial role in crop management and weed control (Flores et al., 2020). This study aims to introduce an optimized target detection algorithm to address the challenge of crop row recognition in complex environments. Drawing inspiration from (Meng et al., 2023), our research focuses on enhancing detection speed and efficiency for single-target detection. Initially, we employed a multi-scale prediction approach, enabling the model to better adapt to targets of varying sizes (Wang C. et al., 2023). This improvement elevated the model’s AP to 92.42%. Subsequently, we integrated ECA and CA visual attention mechanism modules, facilitating the model in more effectively extracting and focusing on target-relevant information while suppressing background noise (Wu et al., 2020). By incorporating a variety of visual attention mechanisms and spatial pyramid pooling modules, our algorithm achieved significant performance enhancements in experimental settings. Compared to previous studies, our model demonstrated superior accuracy in crop row recognition within complex environments, achieving higher detection precision and speed, while also being effectively downsized for deployment on embedded devices. Although this modification slightly reduced the AP , it enhanced the model’s detection accuracy and robustness. Furthermore, we utilized the CIoU loss function, which further improved the model’s target localization accuracy and convergence speed, raising the AP to 92.13%. The optimized loss function aided the model in learning better representations of target features. Ultimately, our model maintained high detection accuracy with a detection speed of 15.6 ms and a model size of merely 18.6MB, making it suitable for real-time detection on embedded devices.

(Hu and Huang, 2021) proposed a method that was reliable under sparse weed distribution and optimal lighting conditions but underperformed in scenarios of heavy coverage and insufficient light. In contrast, our model employs an adaptive Gamma enhancement algorithm, based on Retinex theory, for adaptive illumination adjustment in low-light mazie crop row images, showcasing greater stability in complex environments. Compared to the YOLOX-based blue light weeding robot investigated by (Zhu et al., 2022), our study introduces several improvements, such as a lighting adjustment module to enhance low-light recognition capabilities and the incorporation of attention mechanisms to extract key features. These enhancements significantly improved the model’s recognition accuracy under complex lighting conditions (Liang et al., 2022). study utilized edge detection combined with the Otsu algorithm to identify navigation lines between rows of widely planted cotton. Their method, employing least squares for line fitting in the absence of seeds and ditch interference, was limited to specific cotton plantation structures. In contrast, our approach, based on the enhanced YOLOX-Tiny model, adapts more effectively to various crops and growth stages. We implemented multiple technical improvements such as multi-scale prediction, attention mechanism modules, and loss function optimization, enabling the model to maintain high precision under various lighting conditions. Additionally, compared to the method by (Ruan et al., 2023), which estimated crop row numbers using the DBSCAN algorithm, our research directly utilizes the improved YOLOX-Tiny model for crop localization, followed by least squares fitting for centerline identification, yielding better recognition results in shorter timeframes.

Despite the improved target detection algorithm proposed in this study demonstrating excellent performance in the task of maize crop row recognition, we acknowledge the presence of certain limitations. Firstly, although our model maintains high recognition accuracy across a variety of lighting conditions, its capability under extreme conditions, such as severe occlusion or very low light, still requires enhancement.

Secondly, the experimental dataset utilized in this study is primarily focused on maize crops, which may limit the direct applicability of the model to other row crops. There is a wide variety of row crops, and differences in morphological characteristics, growth stages, and planting densities among crops could impact the adaptability and detection accuracy of the model.

Furthermore, while our approach enhances detection accuracy and speed through the use of lightweight neural networks, the incorporation of various optimization techniques, and the optimization of the model structure, thereby ensuring the model’s practicality and deployability, balancing the complexity of the model with the consumption of computational resources remains a challenge in real-world agricultural applications.

## 5 Conclusions

The improved YOLOX-Tiny model introduces a multi-scale prediction approach, significantly enhancing the model’s adaptability to objects of varying sizes. Experimental outcomes indicate that this strategy elevates the model’s AP to 92.42%. The integration of efficient channel attention and coordinate attention mechanisms concentrates on the pertinent information of the targets while mitigating background distractions. Furthermore, the incorporation of the spatial pyramid pooling fast module at the backbone network’s conclusion bolsters detection precision and robustness. Although these attention mechanisms slightly diminish the AP to 92.15%, they effectively underscore the targets’ critical features, culminating in augmented detection efficacy.

Departing from the conventional loss function, the CIoU loss function is employed to refine the accuracy and hasten the convergence speed of maize crop row localization. The utilization of this optimized loss function boosts the model’s AP . to 92.2%, guiding the model towards superior representations of the target objects and thus improving detection performance.

By pinpointing key points on maize seedlings, our algorithm not only preserves the accuracy of crop row centerline recognition but also considerably reduces the centerline fitting time by 42 ms. The average angle discrepancy between the fitted lines and manual annotations is a mere 0.59°. These experimental findings corroborate the algorithm’s real-time performance and accuracy benefits in extracting navigation lines. The proposed enhanced YOLOX-Tiny model showcases exceptional performance and practical utility in maize crop row detection, excelling in both detection accuracy and speed, alongside a compact model size, making it highly suitable for real-world applications in intelligent weed control machinery navigation.

Despite the notable success of the target detection algorithm based on the improved YOLOX-Tiny model in maize crop row recognition tasks, we are acutely aware of certain limitations inherent in our study, which delineate directions for future work. Firstly, concerning the model’s performance under extreme environmental conditions, we note a performance decline in scenarios of severe occlusion or very low lighting. Future research could delve into more advanced image preprocessing technologies, such as deep learning-based image enhancement algorithms, to ameliorate the quality of input images, thereby augmenting the model’s robustness under adverse conditions. Moreover, exploring novel attention mechanisms may furnish the model with more nuanced feature extraction capabilities, further enhancing its adaptability to complex environments. While our research primarily focuses on maize crops and achieves commendable results, this limits the model’s direct applicability to other row crops. Future work should aim to construct a multi-crop dataset encompassing various types of row crops and diverse growth conditions. Additionally, employing transfer learning and domain adaptation techniques could effectively migrate the model from one crop to another, reducing dependence on extensive labeled data and accelerating the adaptation process for new crops. Lastly, considering the demands for model size and computational efficiency in practical applications, future research should also pay attention to model lightweighting and acceleration techniques. Techniques such as network pruning, quantization, and knowledge distillation could significantly reduce the model’s parameter count and computational requirements, making it more suitable for deployment on resource-constrained embedded devices.

## Data availability statement

The original contributions presented in the study are included in the article/supplementary material. Further inquiries can be directed to the corresponding author.

## Author contributions

HG: Writing – original draft. WZ: Funding acquisition, Methodology, Visualization, Writing – review & editing. XW: Resources, Visualization, Writing – review & editing.
